# On the Nosological Characteristics of Disease

**Published:** 1808-01

**Authors:** 


### Medical and Physical Journal.

VOL. XIX.] JANUARY, 1808. [NO. 107.

*Printed for R. PHILLIPS, by W. Thorne, Red Lion Court, Fleet Street, London*

### *To the Editors of the Medical and Physical Journal.*

GENTLEMEN,

A Friend of mine, not in the profession, lately asked me the following question: "Are there any diseases which are not indicated by external signs? or can a man really suffer corporeal pain, and have at the same time all the criteria, by which physicians are accustomed to judge of diseases in a natural state?" I told him that I could not correctly answer the question, but said that I would consult some who were more capable to give him a satisfactory reply. This I now do, in addressing your correspondents; and I request them to take the subject into consideration, as one not merely of curiosity, or fanciful speculation, but as one of practical importance. The consideration of this subject, I would particularly recommend to that part of the profession who practice in the navy.—In that department, the subjects of practice are men of the lowest class, men subjected to hard labour, and often disliking their situation.—Taking these things into consideration, it will not excite much surprise to learn, that some will prefer the inconvenience of slight disease to their duty; and on the same principle, that they will exaggerate to their medical attendants the violence of their sufferings.—Knowing these to be *facts*, we will readily believe that there will be found some, who, to enjoy ease and indolence, either natural, or rendered dear by severe and lengthened toil, will endeavour to counterfeit disease; will prefer the languor of a sick apartment to labour, and assume the language and the mien of suffering, to escape from duty that is hateful to them.

It may, however, be supposed, that the characters of disease, drawn by so unskilful nosologists, will not be very correct; that in the desire to magnify their pretended sufferings, symptoms so jarring and irrelevant will be mingled as to compose a picture too gross to be imposed upon the practitioner as an original. This must undoubtedly be often the case. Yet there will be found some, who assume symptoms so simple, and adhere to them with such obstinacy as to deceive the most skilful. The most successful class will be those, who, recollecting the character of some previous disease, hold forth their sufferings in all the firmness of nosological correctness. I am persuaded that most naval practitioners must have at some time been deceived in this manner: * — though I am afraid they have been as frequently deceived in pronouncing those whose symptoms bore little proportion to their avowed sufferings, impostors.—A practitioner of any feeling, who sees a man apparently suffering pain, even although all the tests of disease, (as pulse, bowels, tongue, &c.) give no intimation of disorder, will rather be inclined to doubt the extent of his own knowledge, or the perfection of his science, than the assertions of his patient. 'Tis a point of delicacy, and one on which a medical man *might* be forgiven for preferring the opinion of the patient to his own.

The bare suspicion, much more the certainty, of the not unfrequent occurrence of instances of imposture of this nature, ought to be a sufficient incitement to the naval surgeon, to pay more than ordinary attention to the characteristics of disease. The mere probability of the punishment, or acquital of a fellow creature depending on his decision, is a weight that must hang heavy on the mind of the naval surgeon: and doubly heavy on *his*, who is deeply conscious of his own ignorance of nosology. If there be

---

* The humanity and good sense diffused through every sentence of the above anonymous communication, (if the writer's name had been subjoined, it would have done him honour) demands some notice from the Editors. — It is unquestionably true, that there are severe internal diseases, unattended by any external signs, and solely marked by the internal symptom of pain. It is equally true, that every medical practitioner, whether physician or surgeon, should have an education that might qualify him to combine the MORALITY of his profession with his practical and nosological knowledge. No medical man is qualified to practice, without an union of these two requisites. It will be obvious to our readers, that no skulker on board an English ship could ever escape the eye of a surgeon so qualified. The countenance itself, is an index that no skulker can disguise from an attentive observer; but if a surgeon is so ignorant or prejudiced, as never to look his patient steadily in the face while he is examining him and his discipline, gross blunders will soon expose his ignorance, and send him to that situation, in some remote country village, where he has no rival, and where alone he may live without contempt.

be men, (as I am told there are) on whose minds the dread of the contempt or ridicule of their companions, stimulates more powerfully to exertion than humanity or their own conscience, I adjure them by the dread of *that* in doubtful cases, to think more seriously and more feelingly than the surgeon did in the following case.

A seamen, on board one of his Majesty's ships cruizing in the West Indies, applied to the surgeon, complaining piteously of a pain in his shoulder preventing the motions of the arm. He could assign no cause for it; said only that some time ago, *it came of itself*, and has gradually increased to its present violence. No external mark could be discovered, and it being suspected to have arisen from some slight strain, it was ordered to be rubbed with a common liniment. This was continued for two weeks with no relief; blisters were then applied, and kept open for another fortnight. At the end of this time, there still appearing no external mark whatever, and suspicions arising, that the man wished to impose on him, the surgeon ordered him to move his arm before him. The poor fellow entreated him to pardon him, but that he could not move it, it gave him such pain. He was allowed to rest for a few days more, when the arm was forcibly moved by another person. 'Twas in vain that the poor wretch implored them to spare him ;—'twas in vain that the tear started in his eye, and the sweat bedewed his forehead; his master, bold in his fancied knowledge, and resolute in the punishment of what his nosology taught him was imposture, ordered a rope and a weight of 18 pounds to be brought; the one he was ordered to swing, or bear from the other the punishment that his *crime* deserved. He implored, he hesitated; when the rope's-end, laid on with no light hand upon his shoulders, made him seize the weight: scarcely had he freed it from the deck, when pain in his shoulder made him forget the lash, and he threw it down. This was a scene which was exhibited many a day, till sullen resentment getting the better of his patience, gave additional support to the previously formed opinion of the surgeon ; and he was about to be discharged to his duty and to punishment as an impostor, when, to the confusion and remorse of his judges, a fatal evidence appeared against them, to confirm the truth of the patient's assertions, and give the fiat to their ignorance and barbarity. A slight swelling appeared on the part with fluctuation ; the part was laid open, and purulent matter to the extent of nearly two pounds was discharged. The smile of triumph and reproach that lightened in the face of the poor patient, when the matter spouted by the side of the lancet, formed a striking contrast with the shame and confusion of the surgeon. The look he gave the surgeon, while he exclaimed, " Now, Sir," impressed me with a lesson I never can forget; a lesson that will tell me not to put such implicit confidence in the decisions of the schools, as to make me neglect the sufferings of my patient, if they appear severe, even although they should give the lie to all the dogmas of physic.

I am, &c.

F. I. I. G.

His Majesty' Ship ———, at Sea,
*November* 27, 1807.

